# Microbes in reconstructive restoration: Divergence in constructed and natural tree island soil fungi affects tree growth

**DOI:** 10.1002/eap.70007

**Published:** 2025-02-14

**Authors:** Kasey N. Kiesewetter, Amanda H. Rawstern, Eric Cline, Gina R. Ortiz, Fabiola Santamaria, Carlos Coronado‐Molina, Fred H. Sklar, Michelle E. Afkhami

**Affiliations:** ^1^ Department of Biology University of Miami Coral Gables Florida USA; ^2^ South Florida Water Management District West Palm Beach Florida USA

**Keywords:** Everglades, hydrology, microbial ecology, plant–microbe interactions, restoration, restoration ecology, soil microbiomes, tree islands

## Abstract

As ecosystems face unprecedented change and habitat loss, pursuing comprehensive and resilient habitat restoration will be integral to protecting and maintaining natural areas and the services they provide. Microbiomes offer an important avenue for improving restoration efforts as they are integral to ecosystem health and functioning. Despite microbiomes' importance, unresolved knowledge gaps hinder their inclusion in restoration efforts. Here, we address two critical gaps in understanding microbial roles in restoration—fungal microbiomes' importance in “reconstructive” restoration efforts and how management and restoration decisions interactively impact fungal communities and their cascading effects on trees. We combined field surveys, microbiome sequencing, and greenhouse experiments to determine how reconstructing an iconic landscape feature—tree islands—in the highly imperiled Everglades impacts fungal microbiomes and fungal effects on native tree species compared with their natural counterparts under different proposed hydrological management regimes. Constructed islands used in this research were built from peat soil and limestone collected from deep sloughs and levees nearby the restoration sites in 2003, providing 18 years for microbiome assembly on constructed islands. We found that while fungal microbiomes from natural and constructed tree islands exhibited similar diversity and richness, they differed significantly in community composition. These compositional differences arose mainly from changes to which fungal taxa were present on the islands rather than changes in relative abundances. Surprisingly, ~50% of fungal hub taxa (putative keystone fungi) from natural islands were missing on constructed islands, suggesting that differences in community composition of constructed island could be important for microbiome stability and function. The differences in fungal composition between natural and constructed islands had important consequences for tree growth. Specifically, these compositional differences interacted with hydrological regime (treatments simulating management strategies) to affect woody growth across the four tree species in our experiment. Taken together, our results demonstrate that reconstructing a landscape feature without consideration of microbiomes can result in diverging fungal communities that are likely to interact with management decisions leading to meaningful consequences for foundational primary producers. Our results recommend cooperation between restoration practitioners and ecologists to evaluate opportunities for active management and restoration of microbiomes during future reconstructive restoration.

## INTRODUCTION

The Anthropocene is a period marked by human‐driven changes to the environment. While many human modifications to landscapes degrade the environment, the field of restoration seeks to actively modify landscapes to improve the quality of habitats by restoring biodiversity, ecosystem functions, and natural conditions (Genes & Dirzo, 2022; Suding, 2011; Wortley et al., 2013). However, large‐scale restoration and reconstruction of habitats to a natural state can be a daunting goal given the complexity of species interactions and biological processes that occur in a healthy ecosystem. Ecological knowledge can inform these efforts and greatly improve restoration success, leading to increases in plant and animal diversity (Pais & Varanda, 2010; Sansevero et al., 2011), water quality (Pander & Geist, 2013), and human health and happiness (Nghiem et al., 2021). Yet, restoration efforts often fail to consider a key component of ecosystems—microbial communities. Microbes have been described as the “unseen majority” that underpin healthy ecosystems (van der Heijden et al., 2008). They are central players in many community processes and ecosystem functions, such as nutrient cycling, carbon sequestration, and decomposition (Fierer, 2017; Treseder & Lennon, 2015; Wagg et al., 2019) and are vital to the health of both plants and animals (Berendsen et al., 2012; Ezenwa et al., 2012). These interactions are especially important in stressful environments (de Zelicourt et al., 2013). For instance, plant‐associated microbiomes can enhance pathogen defense, nutrient acquisition, and tolerance of challenging conditions (e.g., drought, heavy metal pollution, extreme heat; Dastogeer et al., 2022; Glassman & Casper, 2012; Saia et al., 2014). Therefore, understanding how microbiomes respond to restoration management and their roles in ecosystem functions may lead to better restoration outcomes and more resilient ecosystems.

While consideration of microbiomes in restoration is likely to be adopted as part of best practices in the future, there are still many gaps in our knowledge that make this a challenge (Singh Rawat et al., 2023). One of the critical gaps that must be addressed to fully utilize microbiomes in restoration efforts is how different “forms” of restoration impact microbiomes and their cascading effects on primary producers and ecosystem function. Habitat restoration is often complex and not only includes improving existing natural habitat (e.g., prescribed fires) and revegetating existing degraded land (e.g., reforesting abandoned farmland), but can also depend on building lost habitat features (e.g., rebuilding coral reefs). These different types of restoration objectives can present different challenges for establishing and maintaining a healthy microbiome. It remains largely unknown how microbiota is affected when restoration requires complete rebuilding of a lost feature within a terrestrial landscape. For example, if microbiomes are not actively managed during construction of new habitats (e.g., seeded with natural microbiomes), the initial microbial communities that establish from building materials may be mismatched to their new environmental conditions in terms of both their needs (i.e., ecological niche limitation) and their functional capabilities required for proper ecosystem functioning. Also, reconstructive restoration could combine microbes from distinct communities as building a lost feature may require harvesting construction materials from multiple different sources and environments. The resulting microbial community coalescence (i.e., the combining of once entirely distinct microbial communities) could lead to novel interactions among microbes with cascading consequences for ecosystem functioning that may not be easily predicted from the separate communities (Rilling et al., 2015). Therefore, greater study of microbiomes from constructed habitat features is needed if we hope to effectively understand and integrate microbial communities into reconstructive restoration efforts.

Another critical component of incorporating microbial interactions in reconstructive restoration is understanding how management decisions can impact these complex interactions. It is well established that the context in which microbial interactions take place is critical in determining the outcome of the interactions. For example, plant–microbial interactions are often viewed as lying along a continuum of parasitism (antagonistic) to mutualism (beneficial) where the outcome may depend on a wide range of contexts (such as plant host characteristics, microbe characteristics, soil abiotic and biotic conditions, and other environmental factors; Hoeksema et al., 2010), which increases the difficulty of predicting outcomes of microbial interactions. This difficulty is often increased when applied to restoration sites as they often face more intense, unique, and/or compounded environmental stresses as well as more anthropogenic disturbances that may not be present in a natural, pristine environment (e.g., pollutants, habitat loss/fragmentation, severely altered soil communities; Halme et al., 2013; Kouki et al., 2012; Perring et al., 2015). In addition to these changing conditions, restored areas are impacted by management decisions before, during, and after the restoration occurs. As management will likely entail decisions about soils and plants (e.g., moving soils, adding nutrients, selecting plant species, etc.), consideration of how these contexts will impact expected outcomes of microbial interactions is important for successful long‐term restoration of vegetative structure and ecosystem function.

In this study, we address these gaps by evaluating for the first time how large‐scale habitat construction during restoration impacts fungal communities and how these changes in the microbiome impact the success of important native tree species facing different hydrological management decisions in the iconic and highly imperiled Everglades ecosystem. Once spanning 10,000 km^2^, the Everglades has declined to half of its original size due to many anthropogenic stressors such as habitat destruction and changes to hydrology (Sklar et al., 2001). While most of the Everglades consists of sawgrass marsh and flooded slough, tree islands, aggregations of woody vegetation on elevated peat or limestone, play an outsized role in ecosystem function in this landscape. Specifically, tree islands are nutrient hotspots in an otherwise oligotrophic ecosystem (Meshaka et al., 2002; Wetzel et al., 2005) with up to 100 times more phosphorus than the surrounding wetlands (Wetzel et al., 2009). Unfortunately, tree islands are in serious decline (up to 87% in some areas; Sklar & van der Valk, 2002), making them a priority for reconstructive restoration in the Comprehensive Everglades Restoration Plan (CERP), the largest and most costly restoration effort in North America (Clarke & Dalrymple, 2003). Despite the well‐supported evidence that microbiomes play crucial roles within ecosystems and the growing evidence for their importance in restoration, CERP lacks provisions for investigating how microbes contribute to or are affected by Everglades restoration. To integrate microbes into restoration efforts in both the Everglades and the numerous other imperiled ecosystems around the globe, we must understand how reconstructing features of a landscape, such as tree islands, impact soil microbiomes and whether those differences could potentially affect the success of restoration and management efforts.

To fill this gap, we combine field surveys, fungal microbiome sequencing, and a manipulative experiment to improve our understanding of soil fungal communities from constructed and natural tree islands and how differences in these communities' impact native tree performance under alternative hydrological management strategies proposed for this ecosystem. Specifically, we ask (1) how do fungal taxonomic and functional guild diversity, community composition, and putative keystone species (hub taxa) differ between natural and constructed islands and (2) how do differences in fungal microbiomes between constructed and natural islands affect woody growth, foliar growth, and physiology of native tree species under different hydrological regimes? Taken together, these questions provide insight into how constructing lost features within a landscape can impact soil microbiomes and how those changes could alter plant performance under different proposed hydrological management strategies.

## METHODS

### Study area

The Everglades is a mosaic of habitats, including wet prairies, sawgrass ridges, inundated sloughs, and tree islands that provides crucial ecosystem services, such as carbon sequestration and water, throughout South Florida (Brown et al., 2006; Wetzel et al., 2017). Tree islands, which make up ~4% of the historic Everglades, are aggregations of woody vegetation on elevated peat or limestone (Meshaka et al., 2002; Wetzel et al., 2005) and are nutrient hotspots that are estimated to hold up to two‐thirds of the total phosphorus in the entire system (Wetzel et al., 2009). Unfortunately, when a tree island declines or is lost, it releases the sequestered phosphorus into the otherwise oligotrophic environment.

Additionally, tree islands are shaped by the water flow. Natural tree islands are often tear‐dropped shaped, with the long side running approximately parallel to the water flow. The head (or “front”) of the island is the highest elevation and dominated by vegetation that cannot tolerate prolonged flooding, while the tail (or “back”) of the island, which is directly downstream from the head, has a lower elevation and is dominated by more flood‐tolerant vegetation (Sklar & van der Valk, 2002). Due to seasonal rainfall in the Everglades, tree islands have two hydroperiods. The wet hydroperiod peaks in October/November with high water levels (i.e., water stage) that typically inundate island tails and sometimes even entire islands. The dry hydroperiod usually reaches the lowest water levels in May, and as water levels gradually recede, soil surfaces of many tree islands eventually dry out (Sklar & van der Valk, 2002).

To better understand how hydrological management may affect tree islands and other Everglades habitats, the Loxahatchee Impound Landscape Assessment (LILA) in Palm Beach County, Florida, USA (26.489° N, 80.219° W) was established in 2003 by South Florida Water Management District and Army Corps of Engineers. This ~325,000 m^2^ experimental Everglades landscape contains eight ~2500 m^2^ experimental tree islands where different restoration and management decisions that could shape biodiversity and ecosystem functions in the Everglades can be tested. For instance, previous research has shown that these management decisions can impact microbial community composition (Almeida et al., 2022). These islands were constructed with either peat or limestone cores, covered with a top layer of peat substrate, and then planted with a mixture of 10 tree species found on Everglades tree islands (including the four tree species tested in this study) (Stoffella et al., 2010).

### Field collections of tree island microbiome samples

We collected soils from the 8 LILA constructed tree islands and 14 nearby natural tree islands located in the Water Conservation Area 3A in October 2021 (Appendix S1: Table S1). For each of the 22 islands, we aseptically collected two 50 mL soil samples, one from the head of the island, and another from the tail of the island (with the exception of one natural island where the tail could not be accessed). Additionally, from seven of the natural islands sampled and all eight constructed LILA islands, we collected an additional 5 L of soil to be used as inoculum in our greenhouse experiment. All soils were transported back to the University of Miami (Coral Gables, FL) on ice within the same day of collection. Soils used as inoculum were briefly stored at 4°C during the setup of the greenhouse experiment, and soils for fungal community analysis were stored in a −20°C manual defrost freezer for 3 months prior to DNA extractions.

### Microbiome DNA extractions, library preparation, and sequencing

Total fungal DNA was extracted from 0.25 g of soil from each sample (*N* = 43) using a DNeasy PowerSoil Kit (Qiagen, 47017) following standard manufacturer's protocols for processing soil samples with high water content. Samples were then purified following E.Z.N.A Gel Extraction Kit (D2500‐02; Omega Bio‐tek, Norcross, GA, USA). DNA concentrations were checked with a Qubit 4 Fluorometer (Invitrogen, Q33327). Libraries for 43 fungal communities were prepared using a two‐step dual indexing protocol targeting ITS1/ITS2 amplicons (Gohl et al., 2016) and custom sequencing primers (Revillini et al., 2022). The pool was then sent to the University of Miami Genomics Core for sequencing with the Illumina MiSeq platform (v3, 300 bp paired end). The resulting demultiplexed sequence data were then processed through QIIME2 (v.2023.2) where sequences were denoised using DADA2 (Callahan et al., 2016), low‐quality bases were removed, and paired ends were joined (Bolyen et al., 2019) to generate exact sequence variants (ESVs). Data was normalized using total‐sum scaling where relative abundances were calculated by dividing the number of reads by each sample's library size (Lin & Das Peddada, 2020). Additional quality control of removing ESVs with relative abundances of less than 0.25% was also performed to avoid overinflating diversity due to spurious sequences (Reitmeier et al., 2021). After quality control was completed, we had an average of ~6000 reads per sample. These data were used to run analyses on fungal diversity, richness, community composition, functional groups, and network roles as described in the analysis section below.

### Plant–microbiome experiment setup and data collection

To understand how fungal communities from natural and constructed tree islands and their interactions with hydrological management affected native tree performance, we conducted a factorial greenhouse experiment manipulating microbiome origin (i.e., from a natural vs. constructed tree island), inoculation (live vs. sterilized soil inoculum), and hydrology (unconstrained vs. constrained water regimes) across four tree species commonly found on tree islands—*Eugenia axillaris* (white stopper), *Ilex cassine* (dahoon holly), *Annona glabra* (pond apple), and *Chrysobalanus icaco* (cocoplum). Trees were sourced from a native plant nursery (Indian Trails Native Nursery in Lake Worth, FL) where they were propagated from seeds produced by local Everglades genetic stock (i.e., seedling's parental trees were collected from sites within ~48 km of our focal islands).

For each tree seedling/sapling, we created a microcosm where the tree pot was nested in larger vessels that exceeded the volume of the pot in order to allow water treatment to be manipulated (microcosm design modified from Almeida et al., 2022 to accommodate older/larger trees). To manipulate the fungal communities in each microcosm, we added inoculum (3% by volume of the pot) of live or sterile soil collected from one of 15 islands. Live soil inoculum contained an active fungal community from one of the constructed or natural islands of which we previously sequenced the fungal microbiome (see the section *Microbiome DNA extractions, library preparation, and sequencing* for details; Appendix S1: Table S1). Sterile inoculum were the same soils that were autoclaved three times at 121°C.

Our water treatments simulated two different management practices currently being considered for the Everglades. The first, an “unconstrained” water regime, allows natural water accumulation from precipitation, which leads to increases in water stage (i.e., water level) and in many cases inundation of tree islands during the wet season. The second option, a “constrained” water regime, limits the maximum water depth and duration of inundation through diversion of water away from the natural sheet flow into canals (Harvey et al., 2015), resulting in lower water stage in the wet season than the “unconstrained” treatment and the prevention of island inundation. We simulated this key differential effect of these management plans to evaluate their consequences for plant–microbial interactions. Specifically, we determined the projected water stage for each management plan for each month using data available from LILA (Appendix S1: Figure S1). Combining this information with the average tree island soil surface height and the height of water at the lowest water level during the year (also available from this field site), we calculated the water level in our outer microcosm vessels that would match the unconstrained and constrained stages in the field during the wet part of the hydrograph (when unconstrained management would lead to island inundation and constrained management would not). For these calculations, the soil surface of the microcosm's internal pot was treated as the soil surface of the tree island, and the bottom of the vessel was treated as the depth of the water at the lowest point during the year (which is at the end of the dry season in late May). To determine the exact heights for our treatments, we calculated the proportion of that height difference that would have been flooded for the constrained and unconstrained treatments based on the projected water stage for an island in the field during December (Appendix S1: Figure S1). This makes a good model for the wet period hydrology since the difference in water stages between treatments projected for December is within ~1–2 cm of the average projected difference between treatments across the 6 months of the wet hydroperiod from August to January when the unconstrained treatment is projected to have water level be at or near the island soil surface in the field while the constrained treatment targets reduced water levels well below the island surface (Appendix S1: Figure S1). To enact differences in water stage between treatments, all microcosms were watered the same amount from above (i.e., to simulate equal levels of precipitation) but to obtain the correct water stage for the constrained treatment, excess water was allowed to drain through holes drilled in the sides of the outer vessels (i.e., to simulate diverting water under the constrained management strategy).

Trees were placed in the University of Miami Greenhouse (Coral Gables, FL) where all saplings were allowed to acclimate to greenhouse conditions. After an acclimation period, microcosms were set up and initially watered daily to make sure the vessels were filled to appropriate water levels, then watered every 2 days to maintain water levels. To measure changes in tree performance in response to treatments, we noted survival of each tree every 2 days and recorded trunk diameter and leaf number at 6‐week intervals for 5 months (roughly equivalent to the wet season, which is a major hurdle for new plantings success). Finally, we measured stomatal conductance with the LI‐600 Porometer (LI‐COR Biosciences, Lincoln, NE) on all plants (with at least one healthy leaf) at the end of the experiment (2 weeks after the final performance data collection at 5 months).

### Data analysis

All statistical analysis was conducted with R v4.3.0 (R Core Team, 2023). To characterize differences between the fungal communities of constructed and natural islands, we first calculated Shannon–Weiner diversity and performed linear mixed effect models to determine if fungal diversity differed between island type. Each model had island type (natural vs. constructed) and island location (tail vs. head) as explanatory variables, island as a random effect, and fungal diversity as the response. Additionally, we performed a Permutational multivariate analysis of variance (PERMANOVA in vegan; Oksanen et al., 2019) to determine if fungal community composition was significantly different between island type using both Bray–Curtis (to consider relative abundances) and Jaccard (to consider presence/absence) distance matrices (Appendix S1: Table S2). A paired *t* test was conducted to compare the average relative abundances of the ESVs shared between the natural and constructed tree islands. Finally, we used random forest models and Boruta feature selection (Boruta; Kursa & Rudnicki, 2010) to identify fungal taxa of particular importance for distinguishing between the constructed and natural island types (Kursa & Rudnicki, 2010).

In order to determine how fungal taxa of ecological importance in natural islands fared on nearby constructed islands, we identified influential taxa on natural tree islands using a network analysis approach that has recently been experimentally validated in both lab manipulations (Agler et al., 2016) and in nature (Rawstern et al., 2025). The network was built with FastSpar (v1.0.0) (Watts et al., 2019) using ESVs present in at least 2 of the 27 natural tree island sites and default parameters. In order to identify putative keystone fungi—fungal taxa that have a particularly strong effect on the diversity, composition, and structure of their communities—we identified hub taxa by characterizing the degree centrality of each node in the network (Ma et al., 2016). Degree centrality, which measures how connected a node (i.e., fungal taxa) is to the other nodes in the network (Proulx et al., 2005), was calculated using networkx (Hagberg et al., 2008). Fungi were grouped into central taxa (i.e., highly connected, putative keystone fungi; here nodes in the top 25% of centralities), intermediate taxa (i.e., fungi that have intermediate levels of connectivity such that they are included in the network but are not among the most well‐connected fungi), and finally peripheral taxa (i.e., transient fungi whose occurrence in communities is more stochastic and are excluded from the microbiome network) (Ma et al., 2020; Rawstern et al., 2025). We also used FUNGuild (Nguyen et al., 2016) to characterize the guilds of the fungi in the microbiome network. Only guilds that matched to “probable” or higher were included in the guild analyses. Monte Carlo simulations (Waller et al., 2003) were used to compare the guild match percentages between the constructed and natural tree islands.

To assess how shifts in fungal microbiomes and hydrology affected native trees, we evaluated how island type and fungi's presence affected trunk diameter (woody growth), leaf number (foliar growth), and stomatal conductance (physiological response). For each of these plant metrics, we ran a linear mixed model (lme4; Bates et al., 2015) with explanatory variables of inoculation treatment (sterile vs. live), water treatment (constrained vs. unconstrained), plant species identity, and the first two axes of variation in fungal community composition from the multivariate principal coordinate analysis (PCoA) of fungal community composition (described above). The model also included all possible interactive effects between the inoculation treatment, the water treatment, and plant species identity as well as between those terms and each fungal community composition axis. Island was also included as a random effect (Appendix S1: Table S3). If plant species identity or any interaction with plant species was significant, we ran the same model (without plant species) for each of the four species to gain further insight into how each species responded to changes in fungal community composition of inoculum and hydrology (Appendix S1: Table S4).

## RESULTS

### Constructed tree islands exhibit the same overall fungal diversity and common fungal functional guilds as natural tree islands

**FIGURE 1:**
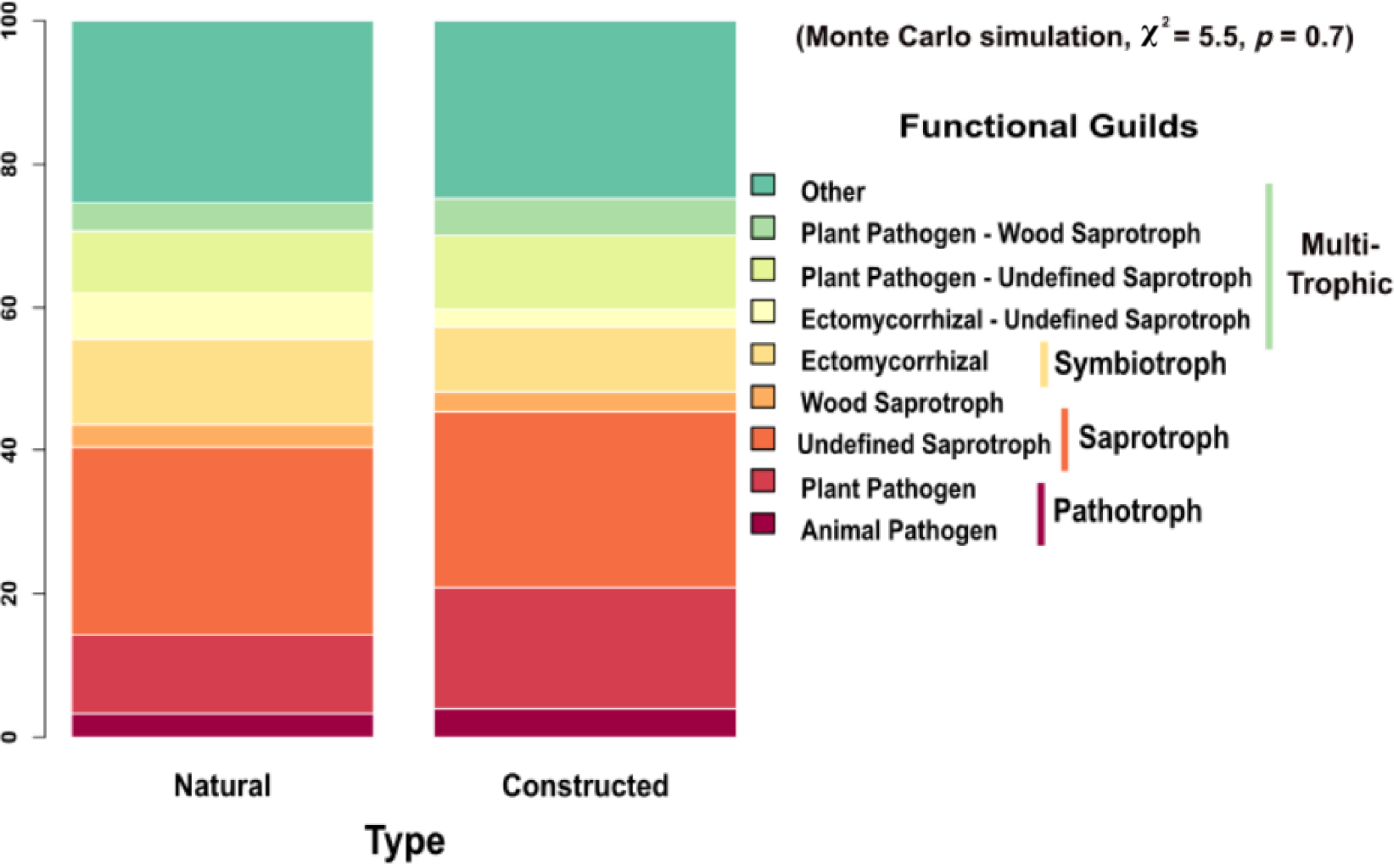
Functional guild distribution does not differ among natural and constructed tree islands. This stacked barplot displays the percentage distributions of top common matched functional guilds among natural and constructed (LILA) tree islands. The key indicates the functional guild while the bold groupings denote the trophic modes. The statistic shown is for a Monte Carlo simulation to compare the frequency distributions between the two habitat types.

There was no difference in overall fungal diversity (means: 2.91 constructed islands, 2.92 natural islands; χ^2^_Type_ = 0.003, *p* = 0.96) or richness (means: 96 constructed, 72 natural; χ^2^_Type_ = 1.37, *p* = 0.24) between the natural and constructed tree islands from the null models. When we examined the top‐most commonly matched guilds, there was no difference between these fungal functional guild percentages among island types (Monte Carlo simulation, χ^2^ = 5.5, *p* = 0.7) (Figure 1). For both tree island types, the trophic mode breakdown was multiple trophic modes (~43%), saprotrophs (~30%), pathotrophs (~15%), and symbiotrophs (~12%) (Figure 1). These results suggest that over relatively short time scales (~18 years) constructed tree islands can accumulate levels of diversity and functional variation within their soil fungal communities similar to natural islands without active restoration of the fungi during island construction.

### Constructed tree islands differ in fungal microbiome composition from natural tree islands and lack many of the natural islands' hub fungi

**FIGURE 2:**
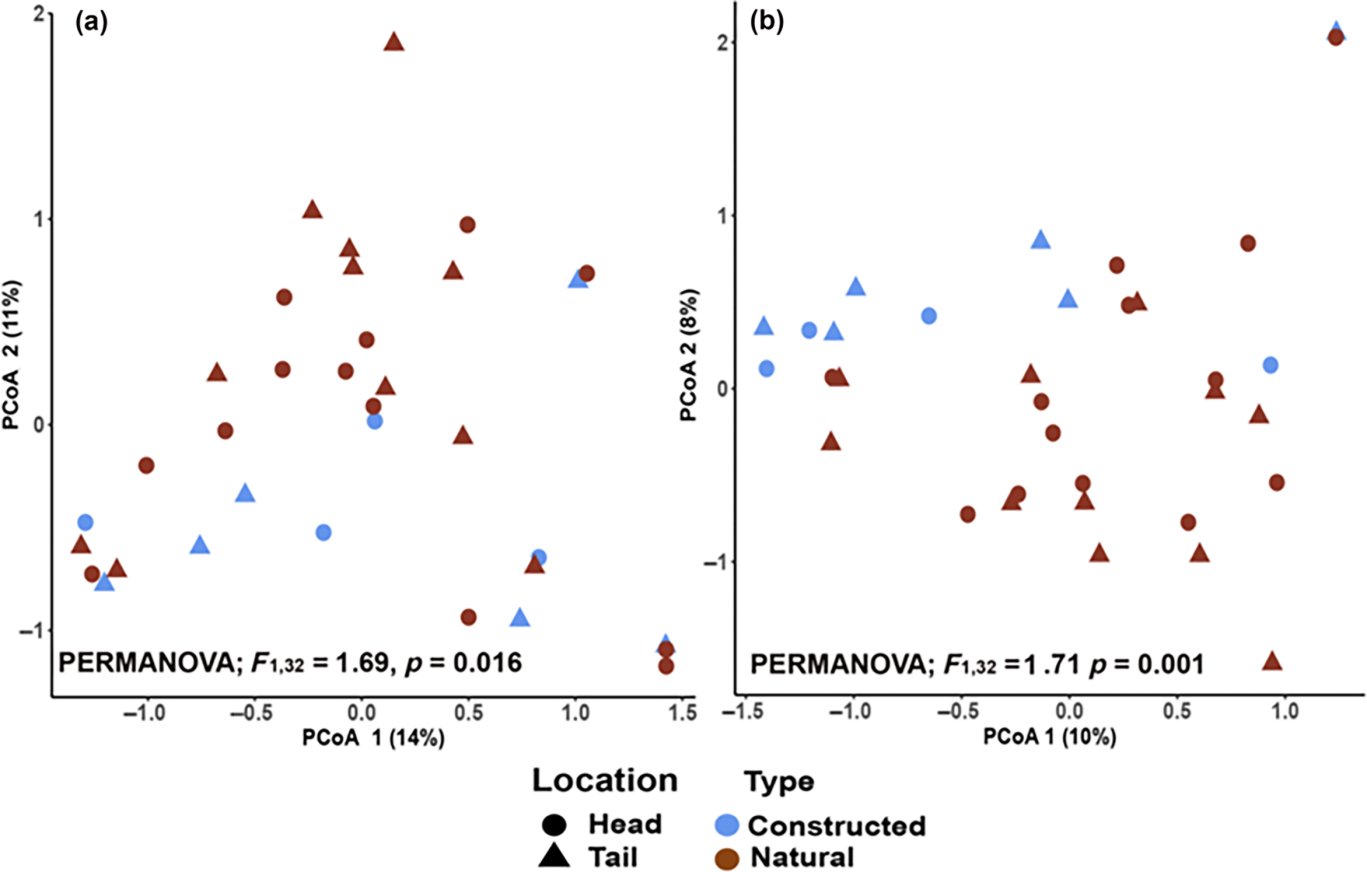
Fungal microbiome compositions of natural and constructed tree islands differ based on changes in relative abundances and present/absence of taxa. Community composition differs by habitat type of natural tree islands and constructed tree islands but not by island location of head or tail in both (a) and (b). The ordination of community composition shown in (a) uses a Bray–Curtis distance matrix, which accounts for the relative abundances of taxa, and the ordination of community composition in (b) uses a Jaccard distance matrix, which accounts only for presence/absence of taxa. Each point represents the fungal community composition of a different tree island at their head and tail locations, and statistics for the PERMANOVA of habitat type are given above each ordination.

We found that fungal microbiome composition significantly differed between natural and constructed tree islands (PERMANOVA; *F*_1,32_ = 1.69, *p* = 0.016) (Figure 2a), suggesting that constructing islands without actively restoring microbiomes does not result in assembly of a natural island fungal microbiome. In particular, the fungal community composition along PCo2 significantly diverged between constructed and natural islands (*t*_33_ = −2.31, *p* = 0.03; Figure 2a). Despite differences in the hydrology of the heads and tails of islands, fungal community composition did not differ between heads and tails (PERMANOVA; *F*_1,32_ = 0.35, *p* = 0.7) (Figure 2a). We found that most of the variance in fungal community composition was attributed to presence versus absence of fungal taxa rather than differences in relative abundances (Figure 2b) (PERMANOVA; *F*_1,32_ = 1.71, *p* = 0.001). We further corroborated this by finding no difference in the average relative abundances of shared taxa between the natural and constructed tree islands (paired *t* test; *t*_116_ = −1.36, *p* = 0.2), indicating that if a microbe was present, it typically made up a similar proportion of the composition in both habitat types. Additionally, utilizing random forest models with Boruta feature selection to identify particularly important fungal taxa in distinguishing the differences between island types, we found seven fungal taxa of interest (Table 1; Appendix S1: Table S5). All of these taxa had a higher relative abundance in constructed islands than in natural islands. Of these seven taxa, four were classified as potential plant pathogens, while only two had a beneficial classification (Table 1). Both of these taxa were classified into two trophic modes, one being symbiotrophic and the other being either pathotroph or saprotroph. These results suggest that potential pathogens may be more influential in structuring the fungal microbiomes of constructed islands than in their natural counterparts.

**TABLE 1:** Microbial taxa identified from random forest models with Boruta feature selection that were particularly important in distinguishing between natural and constructed islands.

Phylum	Class	Order	Family	Genus	Species	Trophic mode	Guild	Citation
Basidiomycota	Agaricomycetes	Polyporales	Ganodermataceae	*Ganoderma*	*eickeri*	Pathotroph Saprotroph	Plant pathogen Wood saprotroph	Gillbertson and Ryvarden (1987), Cannon and Kirk (2007)
Ascomycota	Leotiomycetes	Helotiales	Chaetomellaceae	*Pilidium*	*septatum*	Pathotroph	Plant pathogen	Tedersoo et al. (2014)
Basidiomycota	Agaricomycetes	Polyporales	Ganodermataceae	*Ganoderma*		Pathotroph Saprotroph	Plant pathogen Wood saprotroph	Gillbertson and Ryvarden (1987), Cannon and Kirk (2007)
Ascomycota	Eurotiomycetes	Eurotiales	Aspergillaceae	*Penicillium*	*subarcticum*	Saprotroph	Dung saprotroph Undefined saprotroph Wood saprotroph	Duncan and Eslyn (1966), Bills et al. (2013), Tedersoo et al. (2014)
Ascomycota	Leotiomycetes	Helotiales	Mollisiaceae	*Mollisia*	*albogrisea*	Pathotroph Symbiotroph	Endophyte Plant pathogen	James et al. (2006), Tedersoo et al. (2014)
Ascomycota	Dothideomycetes	Pleosporales	Didymosphaeriaceae	*Paraconiothyrium*	*babiogorense*	Saprotroph	Undefined saprotroph	Tedersoo et al. (2014)
Mortierellomycota	Mortierellomycetes	Mortierellales	Mortierellaceae	*Mortierella*		Saprotroph Symbiotroph	Endophyte Litter saprotroph Soil saprotroph Undefined saprotroph	Cannon and Kirk (2007), Tedersoo et al. (2014)

*Note*: All fungal taxa had higher relative abundance in constructed islands. Trophic mode and guild were determined from FUNGuild.

To gain insight into whether compositional changes affected fungi that are influential in microbiome communities, we used microbiome networks to identify high centrality, “hub” taxa (putative keystone fungi that are likely influential in fungal community dynamics) as well as intermediate taxa (taxa connected within fungal networks and thus likely consistent parts of fungal community) and peripheral taxa (stochastic, transient fungi) within natural tree islands and evaluated their fates on constructed islands. In the natural tree island fungal microbiome network, we identified 95 fungal taxa classified as central (putative keystones), while the remaining 263 fungal taxa within the network were classified as intermediate. Finally, an additional 229 fungi were classified as transient, peripheral taxa (taxa only found at one site across all the native tree islands). The degree centrality of the fungi was Poisson distributed (Shapiro–Wilk, *W* = 0.98), which is indicative of networks with low modular structure (Appendix S1: Figure S2). Plant pathogens and saprotrophs were tied for the most common guild affiliations of the central fungi, suggesting that these functional groups involved in interactions with plants and decomposition may be especially important in structuring fungal communities occurring today on Everglades tree islands. Saprotroph was also the most common guild of the intermediate taxa. Interestingly, ~50% of the central fungi (and ~80% of the intermediate fungi) found in the natural island microbiomes were missing from the constructed tree islands, suggesting that many keystone microbes may be completely absent from constructed islands and that compositional divergence between native and constructed islands includes major changes in the occurrence of influential microbes rather than in transient, stochastic taxa. Overall, these results indicate that despite similar richness and diversity of the fungal community and frequencies of taxa from the most common fungal functional guilds, constructed islands differ substantially in their microbiome composition and lack many putative keystone fungi.

### Divergence in fungal composition between natural and constructed tree island microbiomes explains variation in woody growth of Everglades trees

**FIGURE 3:**
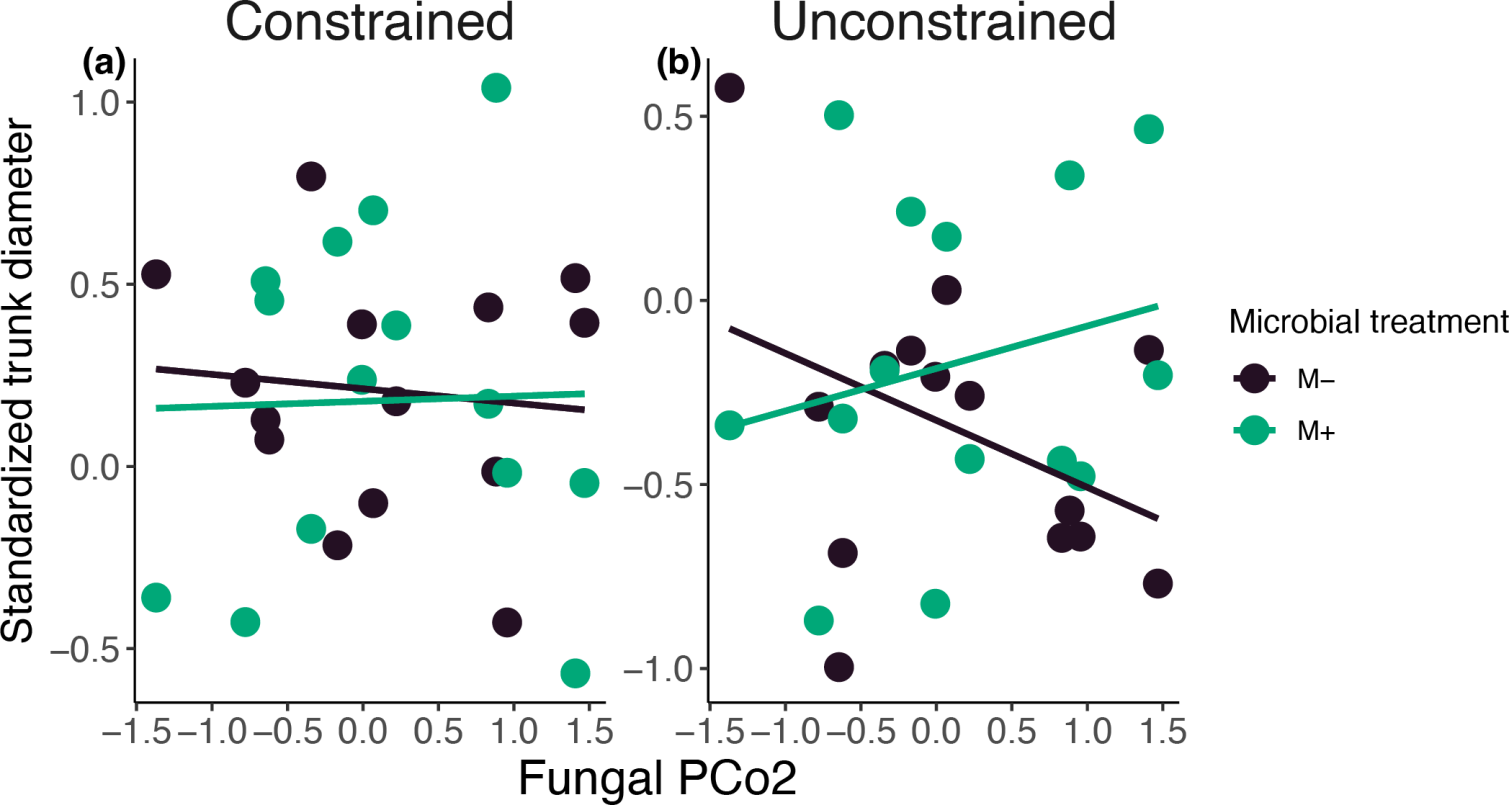
Variation in fungal community composition along PCo2, the microbial inoculation treatment, and the water regime treatment interactively affected woody growth (trunk diameter: χ^2^_1,298_ = 5.83, *p* = 0.016). Shifts in community composition along PCo2 did not strongly affect woody growth in the (a) constrained water treatment but did impact woody growth in the (b) unconstrained water treatment. In the unconstrained water regime, trees grown in the presence of microbiomes with higher values of PCo2, which are found predominately on constructed tree islands, had greater woody growth. M+ corresponds to microbial inoculation treatments where microbes were present (live, microbially active inocula), while M− corresponds to the inoculation treatments where microbes were removed (sterilized, “sham” inocula).

We found that woody growth of trees in our experiment was significantly affected by variation in fungal community composition across the primary axis of community variation that showed divergence between constructed and natural tree island microbiomes (PCo2). Specifically, trunk diameter of the trees at the end our experiment showed a significant three‐way interaction between variation along PCo2, the inoculation treatment, and water treatment (χ^2^_1,298_ = 5.83, *p* = 0.016; Figure 3). When trees experienced the constrained water regime treatment, there were negligible effects of variation across PCo2 on trunk diameter, indicating that the compositional divergence between constructed and natural tree island fungal microbiomes was not important for woody growth when trees experienced constrained water regimes (Figure 3a). In contrast, in unconstrained treatments, variation along PCo2 impacted trunk diameter in the live inoculum treatment, indicating that fungal compositional differences between constructed and natural islands was important for woody growth in more flooded, unconstrained environments (Figure 3b). Trees inoculated with fungal microbiomes with high values of PCo2 had significantly wider trunks in the unconstrained water regime. Taken together with our finding that constructed islands have a shifted fungal community composition along this axis, the link between changes in tree diameter and shifts in composition along PCo2 in unconstrained, but not constrained, hydrology treatments support the conclusion that changes in hydrological management can have different effects on how fungal microbiomes from constructed versus natural islands impact tree growth. Further, as high values of PCo2 were associated with constructed islands, these findings suggest that unconstrained water regimes may favor beneficial effects of fungal microbiomes from constructed tree islands on woody growth.

**FIGURE 4:**
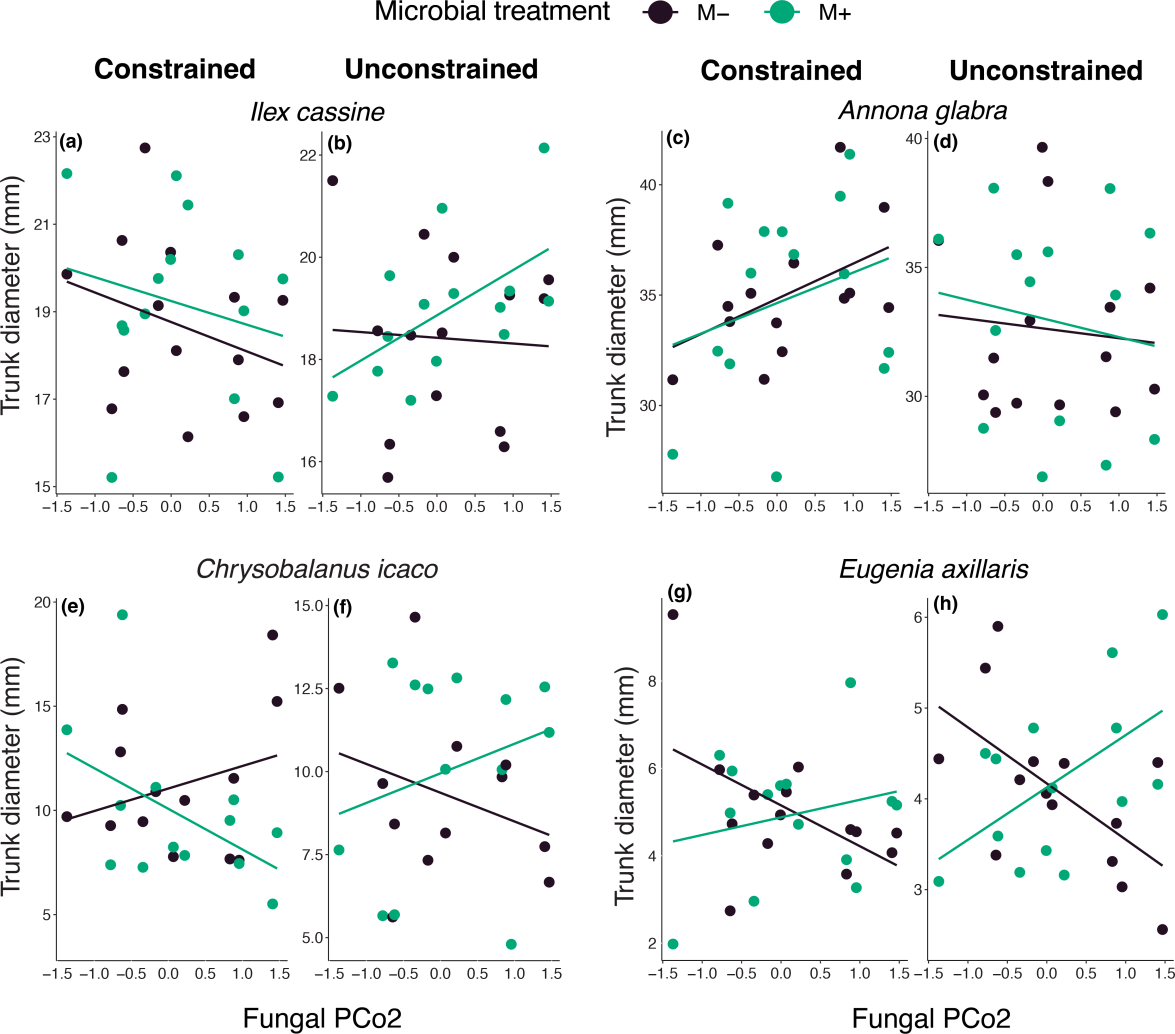
The effects of variation along fungal PCo2 on woody growth for each tree species. (a) For *Ilex cassine* in constrained treatment, woody growth is higher in trees inoculated with microbes (than when microbes are absent), with increasing values of PCo2 having a slight decrease in trunk diameter. (b) When *I. cassine* was grown in unconstrained treatment, the relationship between fungal PCo2 and woody growth showed a trend towards increasing trunk diameter with increasing values of PCo2. In *Annona glabra*, woody vegetation was significantly affected by water regime (χ^2^_1,103_ = 4.05, *p* = 0.044), but not microbial presence or their interaction, as there were generally wider trunk diameters in (c) constrained compared with (d) unconstrained treatments. Woody growth in *Chrysobalanus icaco* significantly responded to the interaction between microbial inoculation, water regime, and variation along PCo2 (χ^2^_1,51_ = 6.11, *p* = 0.013). (e) In constrained treatments when trees were inoculated with microbes, *C. icaco*'s trunk diameter decreased with increasing values of PCo2, while (f) it increased along PCo2 in unconstrained treatments. Woody growth in *Eugenia axillaris* was significantly affected by the interaction between microbial treatment and variation along PCo2 (χ^2^_1,51_ = 5.19, *p* = 0.023), but this did not depend on the water regime. Both the (g) constrained and (h) unconstrained treatments have wider trunks in the live inoculation treatment at high values of PCo2 and wider trunks when microbes are absent from inoculum at low values of PCo2.

Next, while this effect was consistent across species (i.e., no significant interaction of the water regime, fungal composition, and inoculation with tree species identity), other effects depended on tree species identity. For instance, we found a significant interactive effect on trunk diameter of tree species identity with the inoculation treatment and variation along PCo2 (χ^2^_3,298_ = 12.0, *p* = 0.007; Figure 4), indicating that the effects of fungal composition along this axis depended on tree species when water regime is not considered. When evaluating species separately, we found that both *E. axillaris* and *C. icaco* showed a significant interaction which included variation along PCo2 (the axis with significant divergence in fungal composition of constructed and natural islands). *E. axillaris* has higher woody growth when trees are inoculated with fungi from constructed tree islands (higher values of PCo2) regardless of which water regime the trees experienced (inoculation treatment × PCo2: χ^2^_1,72_ = 7.69, *p* = 0.005; Figure 4g,h), while *C. icaco*'s response to the inoculation treatment and variation along PCo2 depended on the water treatment (inoculation treatment × PCo2 × water treatment: χ^2^_1,51_ = 6.11, *p* = 0.013; Figure 4e,f). Like *E. axillaris*, *C. icaco* trees grown with fungal microbiomes with high PCo2 values (which occurred on constructed tree islands) had wider trunks in the unconstrained treatment than trees in the sterile inoculation treatment. In contrast to the other tree species in this study, *C. icaco* had narrower trunks when grown in fungal microbiomes with high PCo2 values in the constrained treatment. These results suggest woody growth of *C. icaco* benefits from inoculation with fungal microbiomes from natural islands if the water regime is constrained and from inoculations from constructed tree islands if the water regime is unconstrained. These findings highlight two important aspects of tree islands fungal microbiomes. First, variation in fungal communities associated with different tree island types is crucial for determining woody growth of tree island species. Second, both abiotic context (here hydrological regime) and biotic context (here species identity) are critical for understanding fungal benefits.

### Unknown environmental factors shape fungal microbiome effects on foliar growth of Everglades trees

Variation across the other primary fungal community composition axis (PCo1) explained changes in foliar growth of the trees in our experiment. In our analysis across all tree species, we found that foliar growth was significantly affected by the interaction between inoculation treatment and variation on PCo1 (χ^2^_1,298_ = 4.96, *p* = 0.026), where trees inoculated with fungal microbiomes with high values of PCo1 had fewer leaves than trees in the sterile (sham) inoculation treatment or trees inoculated with live inoculum with lower PCo1 values. Unlike PCo2, differences in composition along this axis were not associated with island type, indicating that variation in the fungal microbiome on PCo1 is likely due to some other important environmental feature or pressure. Therefore, our results suggest that in addition to island type (which is linked to shifts in the fungal microbiome that are important for woody growth), other environmental factors of importance remain to be discovered, which are crucial to a more wholistic understanding of how changes in fungal microbiomes shape tree performance—especially foliar growth—in the Everglades. We also found a marginally significant three‐way interaction between tree species identity, water treatment, and inoculation treatment on leaf number (χ^2^_3,298_ = 7.14, *p* = 0.068), suggesting that how foliar growth responds to inoculation in a general sense (i.e., regardless of composition of the inoculum) depends on water regime.

**FIGURE 5:**
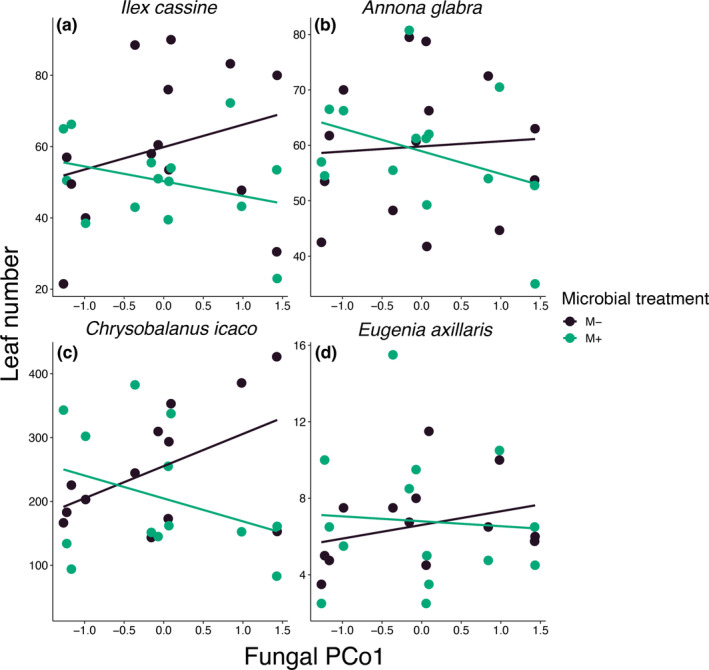
The relationship between variation in fungal community composition along PCo1 and leaf number. (c) Foliar growth (measured here as leaf number) of *Chrysobalanus icaco* significantly responded to the interaction between microbial treatment and variation along PCo1, with trees inoculated with live microbes with high values of PCo1 having reduced foliar growth (χ^2^_1,51_ = 5.49, *p* = 0.019). All other tree species, (a) *Ilex cassine*, (b) *Annona glabra*, and (d) *Eugenia axillaris*, followed the same trend of decreasing foliar growth with increasing PCo1 in the live microbiome treatment.

Because of the interaction with species identity, we next evaluated each tree species separately to determine species‐specific effects of fungal microbiomes and water regimes on foliar growth. We found that the interaction between variation in fungal composition along PCo1 and inoculation affected foliar growth of one species, *C. icaco* (χ^2^_1,51_ = 5.49, *p* = 0.019; Figure 5c). Higher values of PCo1 were associated with decreased foliar growth in the live inoculum treatment, which was the same relationship found in the overall, multi‐species model. Despite *C. icaco* being the only species that had a significant effect of the interaction between variation in PCo1 and inoculation, all species followed the same trend—higher values of PCo1 were associated with lower foliar growth when plant were inoculated with microbes (Figure 5). Therefore, it is the strength, rather than the direction, of the effects that are species‐specific. In addition, these results suggest that there are environmental factor(s) impacting fungal community composition regardless of water regime and thus that some abiotic factors could be incorporated into restoration that would generally increase fungal benefits to foliar growth that would be less dependent on hydrological management.

### Shifts in water regime and fungal composition affect tree physiology

**FIGURE 6:**
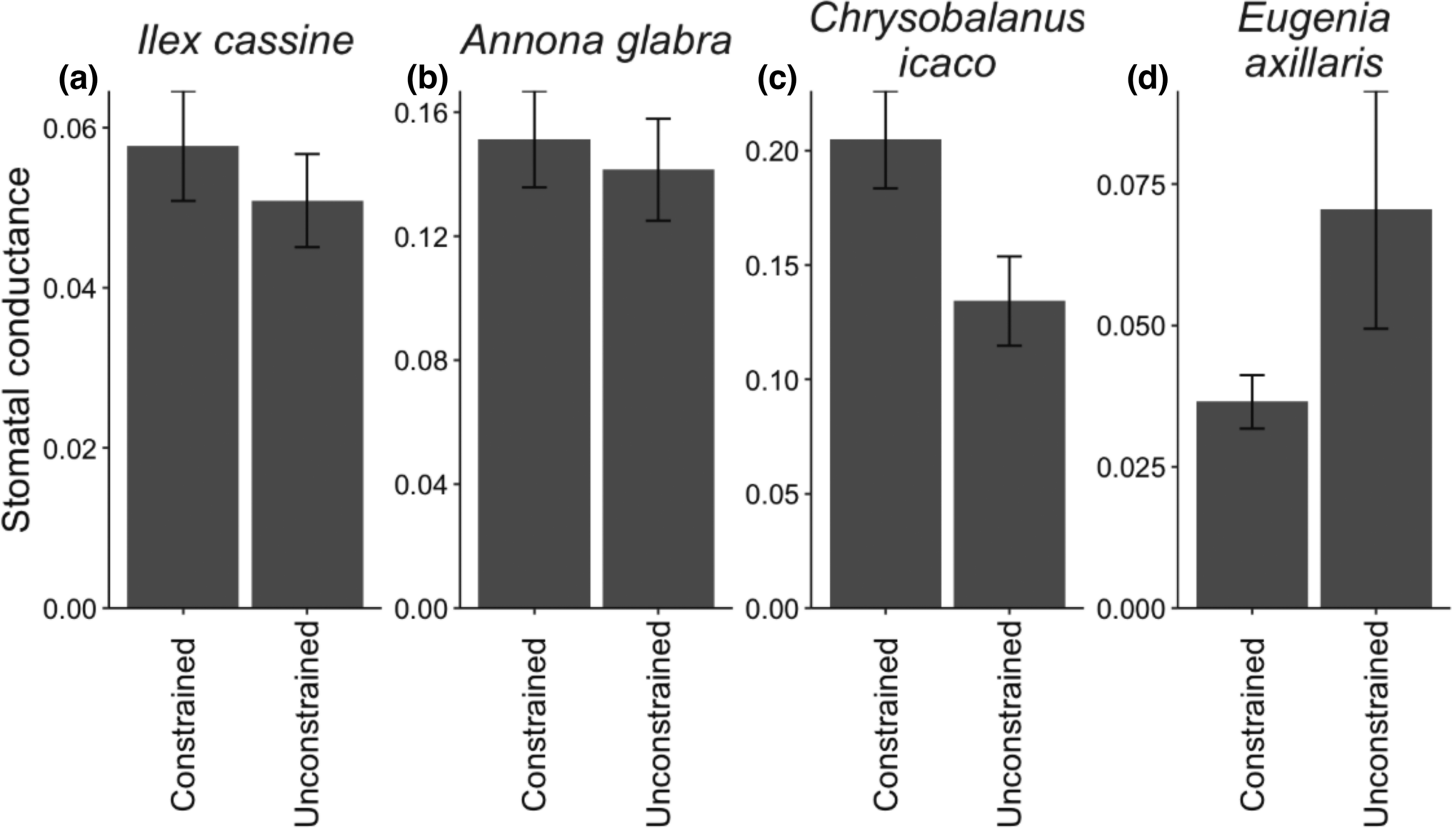
Water regime affected tree physiology in species‐specific ways (significant interaction between species and water: χ^2^_3,298_ = 9.59, *p* = 0.022). In constrained treatments, stomatal conductance trended higher in (a) *Ilex cassine* and (b) *Annona glabra* and was significantly higher in (c) *Chrysobalanus icaco* (χ^2^_1,51_ = 6.33, *p* = 0.012). (d) *Eugenia axillaris* showed the opposite trend, where stomatal conductance was lower in the constrained treatment than in the unconstrained treatment.

Finally, we found that water regime affected an important aspect of tree physiology, stomatal conductance, and that these effects varied among tree species (significant interaction between species and water: χ^2^_3,298_ = 9.59, *p* = 0.022; Figure 6). This significant interaction was largely driven by the contrast between effects found for *E. axillaris*, which trended towards increased stomatal conductance in the unconstrained treatment (Figure 6d), and the other three species, which showed the opposite response (best illustrated by the ~34% decrease in *C. icaco*'s stomatal conductance in the unconstrained treatment; χ^2^_1,51_ = 6.33, *p* = 0.012; Figure 6a–c). In the species‐specific analyses, we also noted that community composition along PCo1 was also important for stomatal conductance in some cases. Stomatal conductance of *A. glabra*, for example, was affected by the interaction between water regime, inoculation, and variation along PCo1 (χ^2^_1,103_ = 3.59, *p* = 0.058). Here, we found that when trees were inoculated with fungal microbiomes with larger values of PCo1, they had lower stomatal conductance in constrained treatments and higher stomatal conductance in the unconstrained treatments. Interestingly, when compared with the effects of these same treatments on foliar growth for *A. glabra*, we find that in the unconstrained treatments stomatal conductance increases and foliar growth decrease when grown in the presence of high PCo1 fungal microbiomes, while in constrained treatments both foliar growth and stomatal conductance decrease when grown with high PCo1 fungal microbiomes. These results illustrate how water regimes may lead to conflicting effects of fungal composition on different aspects of tree performance/function (as seen here with the unconstrained treatment) or agreement in the same fungal composition effects on multiple components of tree function (as seen here with the constrained treatment).

## DISCUSSION

Microbiomes not only provide essential ecosystem functions important for a healthy habitat, but they also underpin natural plant community dynamics in degraded ecosystems (Trivedi et al., 2020). While there has been an emphasis on the need for more in‐depth research evaluating natural microbiomes and how microbiome composition scales up to impact native plants (Farrell et al., 2020), it remains that very few restoration projects consider microbiomes in their management plans (Coban et al., 2022), particularly in reconstructive landscape restoration. Our study used field surveys of both natural and experimental tree islands, microbiome sequencing, and network biology to assess the differences between soil fungal communities of natural and constructed landscape Everglades tree islands to inform future constructed landscape management decisions. In addition, we used manipulative experiments to investigate how different hydrological regimes and microbiome composition between reconstructed and natural habitats interact to impact multiple aspects of native Everglades tree species performance. Our results showed that constructed tree islands can accumulate levels of diversity and functional variation within their soil fungal communities similar to natural islands but lacked many putative keystone fungi that may be important for long term maintenance of microbiome structure and stability. Further, we demonstrated that woody growth, foliar growth, and tree physiology were impacted by complex interactions between the variation in fungal community composition, tree species identity, and hydrological management. Shifts in microbiome composition generally affected woody growth and foliar growth of native trees commonly found on healthy tree islands, while tree physiology was mainly affected by water regime. Of particular interest is that woody growth—as measured by tree trunk diameter—was found to depend on the interaction between hydrology and changes in microbiome composition that diverged between constructed and natural islands, highlighting how not including microbiomes in restoration planning in the past can interact with current management to impact tree performance. Below, we discuss the implications of divergence in constructed tree islands' microbiomes and how active microbiome management may be leveraged to increase natural outcomes for tree island restoration, then conclude with novel areas for future research sparked by this study.

Our study found that constructing tree islands without active restoration of the microbiomes can simultaneously result in communities with some similarities to natural islands for univariate community properties, like richness and diversity, and widely diverging community makeups (a more complex, multivariate property of communities). Interestingly, our results from this reconstructive restoration are similar to trends found in studies investigating the effects of revegetation on soil microbiomes. A recent meta‐analysis found that fungal richness did not significantly differ between restored and natural, reference sites, but these sites significantly differed in fungal community composition (Watson et al., 2022). Combined these findings suggest that both reconstructive and revegetation restoration without active restoration of microbiome may not be sufficient to fully recover a natural fungal community. Essentially, if the appropriate microbial players are missing from the reconstructive materials, then the abiotic conditions and plant community cannot select for them. Our results highlight in two main ways that these compositional differences between tree island type are likely crucial to long‐term restoration success and resilience of these built habitats. First, we found that constructed islands lack the majority of influential hub taxa (i.e., putative keystone microbes) found on natural islands. Hub taxa can be critical for maintaining stability of the soil ecosystem through structuring communities and promoting biodiversity (Banerjee et al., 2018), so while constructed tree island microbiomes may currently have microbes in the same functional guilds as natural islands, and may even be performing some natural functions, these microbiomes are likely to be more susceptible to environmental stresses such as droughts and long periods of flooding, which could disrupt their functionality. This finding emphasizes the need for future work investigating the stability of constructed islands' microbiomes through studies evaluating both microbiome structural stability and their responses to experimental perturbations on constructed islands as well as how effectively new taxa can replace missing keystone fungi's roles in function and stability during constructive restoration. Second, we found that compositional differences between island types significantly affected tree growth, suggesting that differences in fungal composition are, in fact, leading to difference in functional effects on tree health, which is important for primary production, nutrient sequestration on islands, and island soil stability and accretion (Rodriguez et al., 2014; Sklar & van der Valk, 2002). This experimental result showing a functional difference between constructed and natural islands is particularly interesting since the analysis mapping fungal taxa to functional guilds suggested that constructed islands can accumulate a similar functional guild makeup as natural islands. One likely explanation is that the functional guild analysis could not detect difference that exist in the community because FUNGuild (the database used in our functional analysis) was only able to match ~40% of fungal taxa in our study to predicted guilds (a common challenge of this method) and functional guild databases generally cannot account for context dependency that may result in microbes performing different functions in different environmental conditions (another common challenge of this method). Future work using metagenomics and metatranscriptomics to generate more comprehensive functional profiling would be valuable for elucidating which functions can recover without active management and which functions/will require more targeted restoration.

Based on our fungal community data from constructed and natural island, we posit that environmental filtering and dispersal limitation are two important mechanisms structuring fungal communities across this landscape that also have important implications for restoration. Microbiomes can display functional redundancy where taxonomically distinct microbes can exhibit similar functions and can be selected through environmental filtering to fill extant niches (Strickland et al., 2009). This can lead to similar functional distributions across habitats despite microbiome compositional differences which is common in soil microbiomes worldwide (Chen, Jiao, et al., 2020; Chen, Wang, et al., 2020) and has also been found in other systems, such as marine waters (Louca et al., 2016) and bromeliad detritus (Louca et al., 2017). Our result of similar diversity and predicted functional capacity on natural and constructed islands lends some support for this functional redundancy hypothesis (Strickland et al., 2009), suggesting that the environments of constructed and natural tree islands exert many similar filtering pressures on the microbiomes. Our results also suggested that many of the compositional differences between natural and constructed islands could be attributed to presence and absence of certain taxa, including hub taxa, rather than differences relative abundance, suggesting that this system may also be impacted by dispersal limitations. Although tree islands are connected through water flow, it is important to note that the Everglades has extremely slow water velocities (Kushlan, 1991), which decreases the probability of survival during the journey to reach a new island. Even if a microbe can survive in these conditions, they may not move far enough to reach another tree island; this is particularly likely in fungi, which are known to experience dispersal limitation in water movement due to their larger cell sizes than prokaryotes (Chen, Jiao, et al., 2020; Chen, Wang, et al., 2020). Due to potential dispersal limitation within this system, islands made in areas with no or very few remaining tree islands could develop significantly different fungal communities (as shown in the constructed tree islands in our study) then those built within a matrix of existing tree islands. Early planting of foundational tree island plant species inoculated with natural microbial communities or introducing key hub taxa to constructed tree islands, especially those with lower connectivity, may be needed to ensure constructed tree islands receive the keystone taxa necessary for microbiomes community structuring and stability.

Our study also highlights how changes in fungal community composition can interact with management decisions to meaningfully impact foundational primary producers, thus influencing potential success of aboveground restoration efforts. Specifically, we found that differences in community composition driven by island types and other environmental factors were important for both woody and foliar growth. Importantly, these differences were often dependent on hydrological treatments. Given that the Everglades is facing many complex stressors, especially hydrological changes, our work has demonstrated that considering microbiomes in restoring and rebuilding tree islands may be important for constructing more natural and potentially more resilient tree islands. This is supported by recent research that has shown that microbiome stress legacy is likely important in determining how microbiomes' benefit changes under environmental stress (Afkhami, 2023; Allsup et al., 2023). For example, in mangroves inoculation with endophytic microbiomes from high‐salinity environments conferred greater benefits to salt‐stressed mangroves than their freshwater counterparts (Subedi et al., 2022). Additionally, microbiomes from stressful environments provided better relief from drought and temperature stress to tree seedlings experiencing those same stressors in the field compared with their inexperienced microbiome counterparts (Allsup et al., 2023). These results combined with the knowledge that early restoration decisions are important to restoration success (Almeida et al., 2020) and microbial inoculations can be detectable and affect restoration outcomes years later (Neuenkamp et al., 2019), suggest that we need to think more critically about what microbiomes we use in restoration (e.g., matching stress legacy with potential stresses when constructing new tree islands).

In summary, our study highlights how microbiomes can diverge in biologically meaningful ways when new habitats are constructed during restoration and that these differences in the microbiome can interact with current management decisions to impact the success of primary producers. In addition, this work has spotlighted four areas of future investigation that would be especially valuable to increasing our understanding of the complex effects of microbial composition and context dependency needed to successfully utilize microbiomes in rebuilding threatened habitats. First, we were able to identify that differences in island type were driving the divergence along one of the primary axes of variation in fungal community composition, but we were unable to identify what additional environmental drivers were important for distinguishing divergence along the other community composition axis (PCo1)—which we found was important in determining outcomes for foliar growth in native trees. Additional work characterizing abiotic factors that are of known importance for microbial community compositions (e.g., pH and soil nutrients; Fierer, 2017) and biotic factors (e.g., shifts in plant communities; Neuenkamp et al., 2019) would be valuable to future microbiome management of beneficial microbiomes on constructed tree islands. Second, while we saw no significant differences in the functional guilds from the FUNguild analysis, the functions of many species of fungi are uncharacterized in this ecosystem. Therefore, a better functional understanding of entire fungal communities is necessary to understand how changes in composition are driving plant performance. Directly measuring functional traits of (culturable) fungi and hosts (van der Heijden & Scheublin, 2007; Zanne et al., 2020) and leveraging evolving metagenomic and transcriptomic approaches (Chen et al., 2017; Schenk et al., 2012; Streit & Schmitz, 2004; Tringe et al., 2005) are critical next steps in understanding the functional capacity and expressed functional responses to stress in natural and constructed landscapes. Third, investigating what characteristics of natural tree islands result in a stable microbial network of interactions (Hernandez et al., 2021) could be important to understanding what abiotic and biotic factors are driving microbiomes whose functions are resilient and resistant to environmental disturbances. This knowledge could then be used when constructing new islands, targeting features and microbes that would lead to new stable, resilient tree islands. Finally, the knowledge of tree island microbiomes in constructed and natural islands gained from our study emphasizes the importance of future work inoculating newly constructed islands in the field with natural microbial communities that vary in key properties (e.g., origin island's tree composition and hydrology, microbiome composition and network structure, etc.) followed by monitoring of microbiome dynamics and tree performance for improved tree island restoration and understanding microbiome roles in general in reconstructive restoration. Taken together, our study provides evidence that incorporating a microbial perspective into future rebuilding of critical habitat could be a fruitful avenue for building more resilient and productive natural communities.

## AUTHOR CONTRIBUTIONS

Michelle E. Afkhami, Eric Cline, Carlos Coronado‐Molina, Fred H. Sklar, and Kasey N. Kiesewetter contributed to the conceptualization and design of the study. Kasey N. Kiesewetter collected field samples and prepared samples for sequencing, as well as set up the experiment, with significant help from Amanda H. Rawstern and Michelle E. Afkhami, and managed the experiment. Gina R. Ortiz procured/repotted trees and provided care during acclimation prior to the greenhouse experiment. Eric Cline provided management and access to constructed tree islands. Carlos Coronado‐Molina and Fred H. Sklar assisted in field collections and provided access to natural tree islands sites via airboat. Kasey N. Kiesewetter analyzed the majority of the data with significant help from Amanda H. Rawstern for the microbiome network construction and community composition analyses. Michelle E. Afkhami acquired the necessary funding for the projects and mentored participating graduate students. Kasey N. Kiesewetter, Amanda H. Rawstern, and Michelle E. Afkhami wrote the manuscript, and all authors contributed to editing the manuscript.

## CONFLICT OF INTEREST STATEMENT

The authors declare no conflicts of interest.

## Supporting information


Appendix S1:

## Data Availability

Demultiplexed sequencing data and associated metadata are available in the National Center for Biotechnology Information (NCBI) Sequence Read Archive under accession number PRJNA1136446. All experimental data and code (Kiesewetter et al., 2024) are available on Zenodo: https://doi.org/10.5281/zenodo.13772515.
