# Supplementary material for: Microbes in reconstructive restoration: Divergence in constructed and natural tree island soil fungi affects tree growth
Source: Ecol Appl. 2025 Feb 14;35(1):e70007. doi: 10.1002/eap.70007 (PMC11827290; doi:10.1002/eap.70007)
Supplement: Supplementary file 1 — Appendix S1: [file EAP-35-e70007-s001.pdf]

Ecological Applications

Authors: Kasey N Kiesewetter, Amanda H Rawstern, Eric Cline, Gina R Ortiz, Fabiola Santamaria, Carlos Coronado-Molina, Fred H Sklar, Michelle E Afkhami

Title: **Microbes in reconstructive restoration: Divergence in constructed and natural tree island soil fungi affects tree growth**

Supplementary Figures and Tables

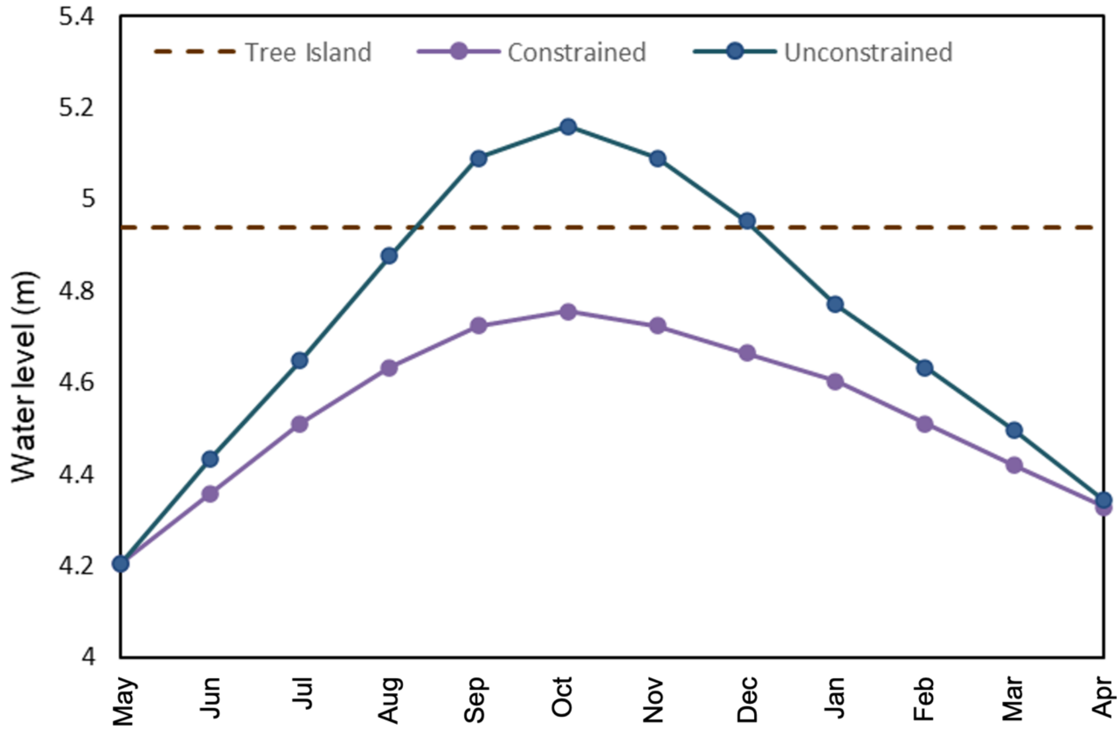

Fig S1. Projected water stage (level) across a year for the constrained and unconstrained management strategies. The blue points and line, which is the unconstrained treatment, shows the projected average water stage each month expected for a tree island if water level was driven exclusively by precipitation (projected based on field data). The purple points and line show the water stage targeted for each month under the constrained treatment, in which water levels during the wet season are constrained to avoid island inundation. Note that water levels are expected to exceed the height of the tree island's soil surface (shown as the brown dashed line) in the precipitation-driven unconstrained treatment (blue line), with water levels peaking in October. In contrast, the constrained treatment (purple line) is never allowed to inundate the islands, which is achieved through water diversion via canals (simulated in our experiment by draining water from microcosm water vessels to appropriate levels).

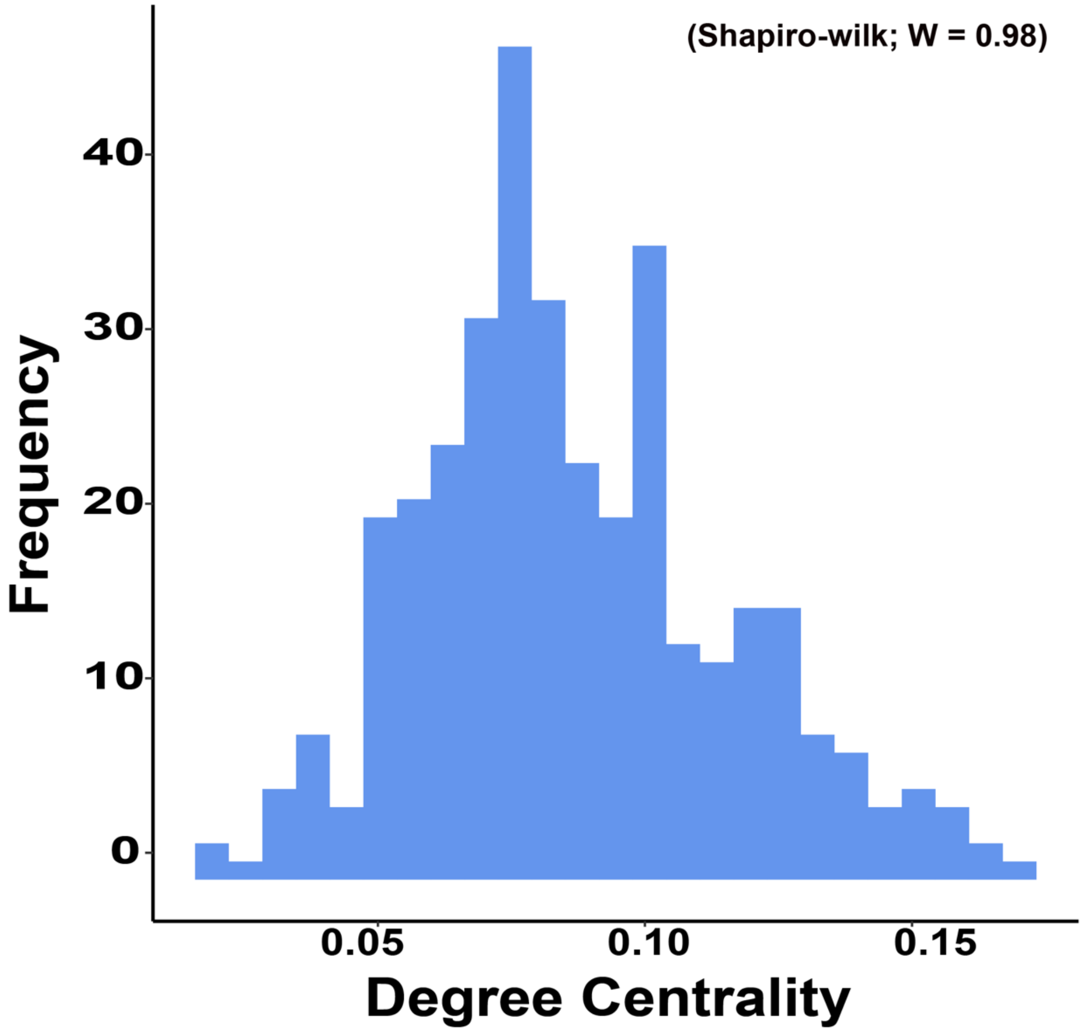

Fig S2. Distribution of degree centralities of natural tree island microbiome network. The degree centrality distribution of the natural tree island fungal network is normally distributed which is indicative of stressed habitats with low modular structure. The x-axis displays the degree centrality value, the y-axis displays the frequency of nodes, and the text states the Shapiro-Wilk normality value.

Table S1. GPS locations of the 14 natural islands within Water Conservation Area 3A where soils were collected.

Island ID	Location	Latitude	Longitude
1	Head	N 25º47'45.3"	W 80º41'22.2"
1	Tail	N 25º46'52.7"	W 80º41'20.2"
2	Head	N 25º47'46.2"	W 80º41'21.5"
2	Tail	N 25º47'45.3"	W 80º41'22.2"
3	*Head*	*N 25º49'13.4"*	*W 80º41'52.1"*
4	Head	N 25º52'52.9"	W 80º40'59.1"
4	Tail	N 25º52'47.3"	W 80º40'58.9"
5	Head	N 25º54'49.9"	W 80º39'19.7"
5	Tail	N 25º54'46.9"	W 80º39'21.3"
6	Head	N 25º53'48.0"	W 80º41'02.6"
6	Tail	N 25º53'45.6"	W 80º41'02.8"
7	Head	N 25º52'17.8"	W 80º41'56.4"
7	Tail	N 25º52'16.3"	W 80º41'57.2"
8	Head	N 25º51'24.3"	W 80º41'51.3"
8	Tail	N 25º51'22.2"	W 80º41'53.6"
10	*Head*	*N 25º46'18.2"*	*W 80º44'09.2"*
10	*Tail*	*N 25º46'18.2"*	*W 80º44'09.2"*
11	*Head*	*N 25º46'52.2"*	*W 80º44'30.0"*
11	*Tail*	*N 25º46'49.8"*	*W 80º44'29.8"*
12	*Head*	*N 25º47'59.8"*	*W 80º44'38.8"*
12	*Tail*	*N 25º47'57.8"*	*W 80º44'39.2"*
13	*Head*	*N 25º48'15.3"*	*W 80º45'19.2"*
13	*Tail*	*N 25º48'11.0"*	*W 80º45'21.4"*
14	*Head*	*N 25º49'06.0"*	*W 80º45'13.7"*
14	*Tail*	*N 25º49'04.3"*	*W 80º45'15.1"*
15	*Head*	*N 25º51'23.6"*	*W 80º46'10.6"*
15	*Tail*	*N 25º51'22.8"*	*W 80º46'11.1"*

NOTE: Islands in italics are those used both for microbiome sequencing and the greenhouse experiments.

Table S2. PERMANOVA results from analyses using both Bray-Curtis (to consider relative abundances) and Jaccard (to consider presence/absence) distance matrices.

	Bray-Curtis			Jaccard		
**Parameters**	**DF**	**F-value**	**p-value**	**DF**	**F-value**	**p-value**
Island Type	1	1.675	**0.016**	1	1.750	**0.001**
Island Location	1	0.848	0.699	1	0.841	0.828
Residual	32			32		

NOTE: Bolded values denotes significant p-values.

Table S3. ANOVA tables for analyses evaluating how microbial treatment (live vs. sterile), water treatment (constrained vs. unconstrained), plant species identity, and the first two axes of variation in the fungal community composition (PCo1 and PCo2) affected trunk diameter (woody growth), leaf number (foliar growth), and stomatal conductance (physiological response).

	Trunk diameter			Leaf number			Stomatal Conductance		
**Parameter**	**DF**	**Chisq**	**p-value**	**DF**	**Chisq**	**p-value**	**DF**	**Chisq**	**p-value**
Species	3	0.961	0.811	3	6.553	0.088	3	1.855	0.603
Water treatment	1	2.687	0.101	1	5.356	**0.021**	1	2.374	0.123
Microbial treatment	1	0.980	0.322	1	3.412	0.065	1	0.177	0.674
PCo1	1	8.810	**0.003**	1	6.618	**0.010**	1	0.625	0.429
PCo2	1	1.106	0.293	1	4.186	**0.041**	1	0.041	0.840
Species*Water treatment	3	0.460	0.928	3	10.683	**0.014**	3	9.594	**0.022**
Species*Microbial treatment	3	1.233	0.745	3	3.876	0.275	3	0.318	0.957
Water treatment*Microbial treatment	1	1.112	0.292	1	1.687	0.194	1	0.000	0.998
Species*PCo1	3	8.735	**0.033**	3	5.335	0.149	3	0.826	0.843
Water treatment*PCo1	1	6.687	**0.010**	1	3.620	0.057	1	0.022	0.883
Microbial treatment*PCo1	1	2.368	0.124	1	4.956	**0.026**	1	0.033	0.856
Species*PCo2	3	11.426	**0.010**	3	3.194	0.363	3	0.585	0.900
Water treatment*PCo2	1	1.632	0.201	1	3.018	0.082	1	0.381	0.537
Microbial treatment*PCo2	1	4.950	**0.026**	1	0.002	0.969	1	0.043	0.836
Species*Water treatment*Microbial treatment	3	0.695	0.874	3	7.139	0.068	3	4.959	0.175
Species*Water treatment*PCo1	3	7.677	0.053	3	5.918	0.116	3	0.006	1.000
Species*Microbial treatment*PCo1	3	1.223	0.747	3	4.142	0.247	3	0.336	0.953
Water treatment*Microbial treatment*PCo1	1	2.354	0.125	1	1.278	0.258	1	0.007	0.936
Species*Water treatment*PCo2	3	4.356	0.225	3	2.585	0.460	3	2.284	0.516
Species*Microbial treatment*PCo2	3	12.030	**0.007**	3	0.834	0.841	3	0.250	0.969
Water treatment*Microbial treatment*PCo2	1	5.826	**0.016**	1	0.145	0.704	1	0.970	0.325
Species*Water treatment*Microbial treatment*PCo1	3	2.207	0.531	3	4.160	0.245	3	2.309	0.511
Species*Water treatment*Microbial treatment*PCo2	3	5.335	0.149	3	0.361	0.948	3	2.247	0.523
Residuals	298			298			298		

NOTE: Bolded values denote significant p-values.

Table S4. ANOVA tables resulting from analyses evaluating how microbial treatment (live vs. sterile), water treatment (constrained vs. unconstrained) and the first two axes of fungal community composition (PCo1 and PCo2) affected trunk diameter, leaf growth, and stomatal conductance for each individual tree species after overall models showed significant interactions with plant species identity.

		Trunk diameter			Leaf number			Stomatal Conductance		
	Parameters	DF	Chisq	p-value	DF	Chisq	p-value	DF	Chisq	p-value
Ilex cassine	Water treatment	1	1.585	0.208	1	4.335	**0.037**	1	0.450	0.502
	Microbial treatment	1	0.263	0.608	1	0.167	0.683	1	0.771	0.380
	PCo1	1	0.296	0.586	1	0.103	0.748	1	0.110	0.741
	PCo2	1	1.444	0.230	1	0.784	0.376	1	0.760	0.383
	Water treatment*Microbial treatment	1	0.091	0.763	1	2.946	0.086	1	0.197	0.657
	Water treatment*PCo1	1	0.015	0.903	1	0.237	0.626	1	0.065	0.798
	Microbial treatment*PCo1	1	1.012	0.314	1	1.615	0.204	1	0.001	0.974
	Water treatment*PCo2	1	0.573	0.449	1	0.046	0.830	1	0.615	0.433
	Microbial treatment*PCo2	1	0.120	0.729	1	0.748	0.387	1	0.002	0.967
	Water treatment*Microbial treatment*PCo1	1	0.806	0.369	1	0.430	0.512	1	0.000	0.984
	Water treatment*Microbial treatment*PCo2	1	0.270	0.603	1	0.026	0.871	1	0.163	0.686
	Residuals	72			72			72		
Annona glabra	Water treatment	1	4.048	**0.044**	1	0.004	0.951	1	0.173	0.677
	Microbial treatment	1	0.017	0.895	1	1.421	0.233	1	1.144	0.285
	PCo1	1	0.740	0.390	1	0.105	0.746	1	0.002	0.967
	PCo2	1	3.066	0.080	1	0.534	0.465	1	0.002	0.963
	Water treatment*Microbial treatment	1	0.290	0.591	1	2.748	0.097	1	1.919	0.166
	Water treatment*PCo1	1	0.824	0.364	1	0.055	0.814	1	2.480	0.115
	Microbial treatment*PCo1	1	2.627	0.105	1	0.779	0.377	1	1.659	0.198
	Water treatment*PCo2	1	2.038	0.153	1	0.701	0.402	1	0.018	0.894
	Microbial treatment*PCo2	1	0.091	0.764	1	0.085	0.770	1	0.188	0.665
	Water treatment*Microbial treatment*PCo1	1	0.794	0.373	1	0.005	0.944	1	3.593	0.058
	Water treatment*Microbial treatment*PCo2	1	0.008	0.930	1	1.575	0.210	1	0.049	0.826
	Residuals	103			103			103		
Chrysobalanus icaco	Water treatment	1	2.819	0.093	1	5.936	**0.015**	1	0.335	**0.012**
	Microbial treatment	1	1.028	0.311	1	3.779	0.052	1	0.283	0.595
	PCo1	1	9.241	**0.002**	1	7.338	**0.007**	1	1.572	0.210
	PCo2	1	1.160	0.282	1	4.652	**0.031**	1	0.069	0.793
	Water treatment*Microbial treatment	1	1.166	0.280	1	1.869	0.172	1	0.016	0.899
	Water treatment*PCo1	1	7.014	**0.008**	1	4.006	**0.045**	1	0.015	0.902
	Microbial treatment*PCo1	1	2.484	0.115	1	5.490	**0.019**	1	0.113	0.737
	Water treatment*PCo2	1	1.712	0.191	1	3.340	0.068	1	0.014	0.907
	Microbial treatment*PCo2	1	5.193	**0.023**	1	0.002	0.968	1	0.516	0.472
	Water treatment*Microbial treatment*PCo1	1	2.469	0.116	1	1.414	0.234	1	0.012	0.914
	Water treatment*Microbial treatment*PCo2	1	6.112	**0.013**	1	0.160	0.690	1	1.273	0.259
	Residuals	51			51			51		
Eugenia axillaris	Water treatment	1	5.564	**0.018**	1	0.787	0.375	1	2.547	0.111
	Microbial treatment	1	0.060	0.807	1	0.144	0.704	1	0.758	0.384
	PCo1	1	0.011	0.916	1	0.072	0.788	1	0.037	0.848
	PCo2	1	6.408	**0.011**	1	6.230	**0.013**	1	1.521	0.218
	Water treatment*Microbial treatment	1	0.001	0.973	1	0.172	0.679	1	1.807	0.179
	Water treatment*PCo1	1	0.282	0.596	1	1.983	0.159	1	0.072	0.788
	Microbial treatment*PCo1	1	0.086	0.769	1	0.162	0.687	1	0.424	0.515
	Water treatment*PCo2	1	0.578	0.447	1	2.282	0.131	1	0.822	0.365
	Microbial treatment*PCo2	1	7.961	**0.005**	1	1.211	0.271	1	1.056	0.304
	Water treatment*Microbial treatment*PCo1	1	0.173	0.677	1	2.392	0.122	1	0.004	0.952
	Water treatment*Microbial treatment*PCo2	1	0.264	0.608	1	0.526	0.468	1	1.290	0.256
	Residuals	72			72			72		

NOTE: Bolded values denotes significant p-values.
